# Pursuit of Gene Fusions in Daily Practice: Evidence from Real-World Data in Wild-Type and Microsatellite Instable Patients

**DOI:** 10.3390/cancers13133376

**Published:** 2021-07-05

**Authors:** Enrico Berrino, Alberto Bragoni, Laura Annaratone, Elisabetta Fenocchio, Fabrizio Carnevale-Schianca, Lucia Garetto, Massimo Aglietta, Ivana Sarotto, Laura Casorzo, Tiziana Venesio, Anna Sapino, Caterina Marchiò

**Affiliations:** 1 Pathology Unit, Candiolo Cancer Institute, FPO-IRCCS, 10060 Candiolo, Italy; enrico.berrino@ircc.it (E.B.); alberto.bragoni@ircc.it (A.B.); laura.annaratone@ircc.it (L.A.); ivana.sarotto@ircc.it (I.S.); laura.casorzo@ircc.it (L.C.); tiziana.venesio@ircc.it (T.V.); anna.sapino@ircc.it (A.S.); 2 Department of Medical Sciences, University of Turin, 10124 Turin, Italy; 3 Medical Oncology Unit, Candiolo Cancer Institute, FPO-IRCCS, 10060 Candiolo, Italy; elisabetta.fenocchio@ircc.it (E.F.); fabrizio.carnevale@ircc.it (F.C.-S.); lucia.garetto@ircc.it (L.G.); massimo.aglietta@ircc.it (M.A.); 4 Department of Oncology, University of Turin, 10124 Turin, Italy

**Keywords:** gene fusions, *NTRK* genes, next generation sequencing, gene panels, agnostic biomarker, precision medicine, colorectal carcinoma, lung adenocarcinomas, melanoma, immunohistochemistry

## Abstract

**Simple Summary:**

The main aim of our study is to provide real-world data on the enrichment of gene fusions in patients affected by colorectal carcinomas, melanomas, and lung adenocarcinomas characterized by the absence of mutations in the main driver genes and in colorectal tumours with microsatellite instability, using a comprehensive method. By demonstrating this enrichment in a “real-world” cohort, we confirm the feasibility of this approach, suggesting a workflow applicable in diagnostic practice. A second aim points towards a thorough investigation of *NTRK* gene fusions detected in the study by applying different techniques. We therefore provide comparative data across in situ methods and in vitro nucleic acid-based assays to document effective *NTRK* gene fusion detection.

**Abstract:**

Agnostic biomarkers such as gene fusions allow to address cancer patients to targeted therapies; however, the low prevalence of these alterations across common malignancies poses challenges and needs a feasible and sensitive diagnostic process. RNA-based targeted next generation sequencing was performed on 125 samples of patients affected either by colorectal carcinoma, melanoma, or lung adenocarcinoma lacking genetic alterations in canonical driver genes, or by a colorectal carcinoma with microsatellite instability. Gene fusion rates were compared with in silico data from MSKCC datasets. For *NTRK* gene fusion detection we also employed a multitarget qRT-PCR and pan-TRK immunohistochemistry. Gene fusions were detected in 7/55 microsatellite instable colorectal carcinomas (12.73%), and in 4/70 of the “gene driver free” population (5.71%: 3/28 melanomas, 10.7%, and 1/12 lung adenocarcinomas, 8.3%). Fusion rates were significantly higher compared with the microsatellite stable and “gene driver positive” MSKCC cohorts. Pan-TRK immunohistochemistry showed 100% sensitivity, 91.7% specificity, and the occurrence of heterogeneous and/or subtle staining patterns. The enrichment of gene fusions in this “real-world” cohort highlights the feasibility of a workflow applicable in clinical practice. The heterogeneous expression in *NTRK* fusion positive tumours unveils challenging patterns to recognize and raises questions on the effective translation of the chimeric protein.

## 1. Introduction

Practicing precision medicine in oncology demands a detailed diagnostic process for cancer patients, which is particularly valuable for patients at an advanced disease stage. In this scenario, the diagnostic work-up requires a combination of in situ techniques (immunohistochemistry and in situ hybridization) with molecular profiling by in vitro nucleic acid-based assays. In solid tumours, a series of targets that are crucial for first line treatment choice in the metastatic setting, such as *EGFR* mutations, *ALK*, *ROS1* fusions in lung adenocarcinomas, *BRAF* mutations in melanomas, and *KRAS* mutations in colorectal carcinomas, have demanded the introduction of clinical routine investigation of a minimum set of genes into the diagnostic practice [1,2,3]. Over the past years, the use of massively parallel sequencing (aka next generation sequencing (NGS)) in the context of clinical sequencing programs has enabled the identification of novel putative therapeutic targets, some of which have been observed across different malignancies, regardless of the site of origin [4,5,6,7,8]. The latter observation has led to the concept of the histology-agnostic biomarker, which is currently guiding a paradigm shift in reasoning about therapeutic options for oncological patients. Importantly, drugs targeting some of these markers have demonstrated impressive responses in clinical trials, and approvals from regulatory agencies are available or currently in process [9,10,11]. Among these, genomic rearrangements inducing the generation of chimeric transcripts (i.e., “fusion genes”) have acquired a central role among tumour agnostic biomarkers [12,13]. Recent studies identified a 3% to 6% frequency of oncogenic and/or druggable gene fusions in cohorts of patients affected by solid tumours when using a high throughput sequencing approach [14,15].

This scenario is reshaping molecular diagnostics, which has to counterbalance the efficacy and cost effectiveness of testing strategies. Indeed, the main challenges for molecular pathology laboratories are represented by the low prevalence of certain genetic alterations, the need to use inclusive detection methods that allow for high sensitivity, and the demand to modulate current testing modalities to integrate gene fusion detection. Recent studies have documented enrichment of gene fusions in tumours lacking canonical driver mutations and/or harbouring microsatellite instability (MSI), at least in the context of colorectal cancer [16,17,18,19,20]. In this study, we sought to evaluate the added value in the daily practice of pursuing the detection of gene fusions involving druggable genes in patients affected by tumours either with a wild-type result in the main gene drivers assessed in diagnostic practice according to the currently available guidelines and/or harbouring an MSI-high status. We adopted a targeted NGS approach with a panel including 14 genes of interest, which enabled the investigation of recent FDA/EMA approvals for gene fusions. We reported on the feasibility and effectiveness of the methodology, thus providing real-world data on gene fusion testing strategies.

## 2. Materials and Methods

### 2.1. Study Design

The study was carried out on a consecutive series of patients affected by sporadic colorectal cancer (CRC), lung adenocarcinoma (LAC), or melanoma (MEL), who underwent molecular diagnostic analysis on formalin fixed paraffin embedded (FFPE) tissue at our institution (FPO-IRCCS Candiolo Cancer Institute, Candiolo, Italy) between 2015 and 2020.

**Figure 1:**
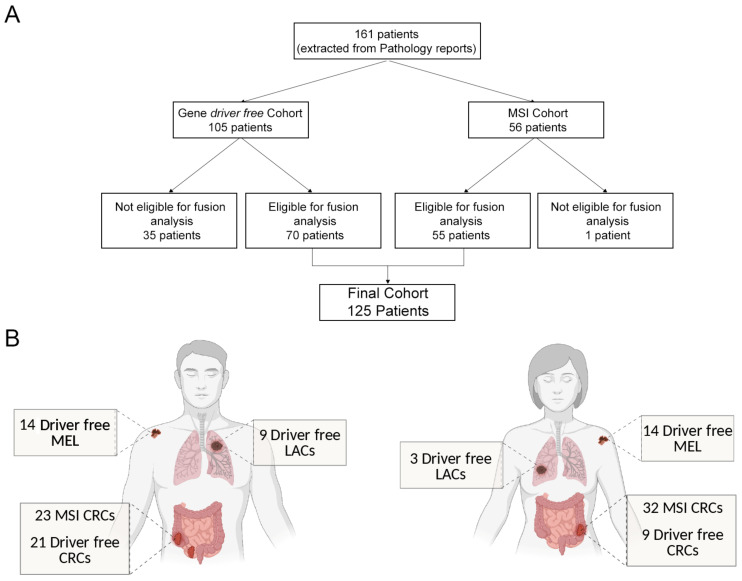
Representation of the “real-world” cohort of the study. (**A**) From an initial population of 161 patients, the histopathological review excluded samples without sufficient tumour cell content. Seventy tumour samples “gene driver free” and 55 MSI cases were eligible for RNA extraction and analysis, leading to a final cohort of 125 patients. (**B**) Final cohort composition: number of melanoma (MEL), colorectal cancer (CRC), and lung adenocarcinoma (LAC) patients according to gender, subdivided in “gene driver free”, and microsatellite instable (MSI) patients.

The cohort selection for the fusion evaluation was carried out by interrogating the pathology reports with the following stringent inclusion parameters: (1) *KRAS*/*NRAS*/*BRAF* wild-type CRCs; (2) LACs not harbouring *EGFR*, *KRAS*, *NRAS*, *BRAF*, and *ERBB2* mutations, and/or *ALK*, *ROS1*, *RET* rearrangements; (3) *BRAF*/*NRAS* wild-type MELs; and (4) CRCs showing microsatellite instability. Tumours meeting the criteria described in 1–3 were labelled as part of the “gene driver free” cohort. A series of 161 candidate patients were identified. Archival haematoxylin and eosin (H&E) slides corresponding to the leftover tissue following the diagnostic analysis were reviewed by a pathologist (CM). Following the review of the tissue sample availability and tumour cell content, the final cohort was composed of 125 cases (Figure 1). The molecular diagnostic analysis originally performed included the sequencing of hot-spot regions of different genes (Myriapod Colon and Lung status kits, Diatech Pharmacogenetics, Jesi, Italy) on the MassARRAY System (Agena Bioscience, Hamburg, Germany). CRCs and MELs were investigated for hotspots in the *KRAS*/*BRAF*/*NRAS* genes; LACs were investigated for hotspots in five genes (*EGFR*/*KRAS*/*NRAS*/*BRAF*/*ERBB2*). *ALK* and *ROS1* fusions for diagnostic purposes were scored negative by immunohistochemistry (IHC), with the VENTANA ALK (D5F3) CDx Assay (Roche-Ventana, Tucson, AZ, USA) and with the anti- ROS1 antibody clone D4D6 (Cell Signalling, Danvers, MA, USA; dilution 1:100, antigen retrieval with the BOND Epitope Retrieval Solution 2, pH 9.0, for 30 min). *RET* fusions were analysed using the EasyPGX ready *ALK*, *ROS*, *RET*, and *MET* qRT-PCR kit (Diatech Pharmacogenetics). CRCs were assessed for microsatellite status using the MSI analysis system v1.2 panel (Promega Corp, Madison, WI, USA), and the products were analysed by capillary electrophoresis using an ABI 3100 Genetic Analyzer (Applied Biosystems, Foster City, CA, USA). The samples were considered to be MSI when harbouring at least two unstable loci [10].

### 2.2. RNA Extraction

RNA was extracted from 7-μm-thick, mesodissected FFPE tissue sections using the Maxwell^®^ RSC RNA FFPE Kit (Promega) and quantified by Nanodrop 1000 and Qubit (Thermo Fisher Scientific, Waltham, MA, USA) assays. Quality was assessed with the Archer Pre-Seq RNA quality control (QC) qPCR Assay (ArcherDX, Boulder, CO, USA), with a threshold Cq < 31.

### 2.3. NGS-Based Fusion Transcript Identification

RNA-based NGS was performed with the Archer FusionPlex Lung panel (ArcherDX), comprising 14 genes (*ALK* LRG_488, *BRAF* LRG_299, *EGFR* LRG_304, *FGFR1* LRG_993, *FGFR2* LRG_994, *FGFR3* LRG_1021, *KRAS* LRG_344, *MET* LRG_662, *NRG1* NM_013956, *NTRK1* LRG_261, *NTRK2 NM_001007097*, *NTRK3 NM_001007156*, *RET* LRG_518, and *ROS1* LRG_997). The selection of the panel was based on the feasibility of the library preparation and on the coverage of the kinase coding-genes with the greatest prognostic and predictive impact with respect to the malignancies included in the study. After cDNA synthesis end repair and A-tailing, a compatible IonTorrent (Thermo Fisher Scientific) adapter was added to each sample. Two anchored-PCR processes amplified the targets and their flanking regions, allowing for the identification of both original transcripts, known fusions, and non-canonical fusion partners [21]. Libraries were quantified and diluted at 50 pM for sequencing on IonTorrent GeneStudio S5 Plus (Thermo Fisher Scientific) for an expected depth of at least 500,000 reads/sample. The Archer Analysis suite (version 6.2) was exploited for the QC and fusion analysis. Samples with fusion QC unique start sites <10 and total reads <500,000 were re-sequenced, and the UBAM was merged with Samtools [22]. Only “strong evidence” fusions were considered as positive, by applying stringent default filters, as follows: (i) reads that support the fusion ≥10; (ii) “fusion_percent_of_GSP2_reads”, i.e., number of breakpoint reads that support the fusion/total number of reads spanning the breakpoint ≥10%; and (iii) “min_unique_start_sites_for_strong_fusion” ≥10. Investigators were blinded to the IHC and qRT-PCR results when assessing the data.

### 2.4. qRT-PCR for *NTRK* Genes

The qRT-PCR Easy PGX *NTRK* (Diatech Pharmacogenetics), which allowed for identifying a total of 32 known *NTRK1/2/3* gene fusions, was run from 200 ng of RNA. Data were analysed with AriaDx software v 1.4 (Agilent, Santa Clara, CA, USA) and were interpreted using the EasyPGX^®^ Analysis Software v.4.0.9 (Diatech Pharmacogenetics). Investigators were blinded to the NGS and IHC results when assessing the data outputs.

### 2.5. IHC

Three μm-thick tissue sections were stained using the pan-TRK assay, clone EPR17341, in the form of the CE-IVD/class I US analytical assay by Roche-Ventana and the RUO antibody by Abcam. IHC reactions were performed on a Ventana BenchMark Ultra Autostainer (Roche Diagnostics, Tucson, AZ, USA). For the Roche pan-TRK assay, we followed the manufacturer’s instructions. The RUO antibody was diluted 1:500 and antigen retrieval Ultra Cell Conditioning 1 (CC1) for 64 min was used. On each slide an external control was included (KM12 cell block sections). Each immunohistochemical run included negative controls with the use of a negative reagent control only. IHC scoring was performed according to the European Society for Medical Oncology (ESMO) Translational Research and Precision Medicine Working Group recommendations [9]. Investigators were blinded to the NGS and qRT-PCR results when assessing the IHC reactions.

### 2.6. Fluorescence In Situ Hybridization (FISH) Analysis

FISH was performed on 5 μm-thick sections of FFPE samples corresponding to the three cases identified as *NTRK* fusion positive by NGS, qRT-PCR, and IHC. FISH was used with a confirmatory approach intent; hence, investigators were not blinded to other data outputs when assessing FISH. The ZytoLight SPEC *NTRK1* or *NTRK3* Dual Color Break-Apart Probes (Zytovision, Bremerhaven, Germany) were used following the manufacturer’s instructions. Scoring was performed on multiple areas (range of 10–38) and was acquired at 40× with the Metafer scanning system (MetaSystems srl, Milan, Italy) and Axio Imager epifluorescence microscope (Carl Zeiss, Oberkochen, Germany). Multiple fields were scanned as the IHC reactions showed heterogeneous patterns of TRK expression. Isis TissueFISH imaging software (MetaSystems srl) was used to analyse the *NTRK* rearrangements, with review by two independent observers with expertise in tissue-based FISH analysis (LC/CM).

### 2.7. In Silico Analyses

“Gene driver positive” control population was selected in silico by interrogating two independent cohorts of patients, accessed through the cBioPortal website [23,24]. We interrogated the Memorial Sloane Kettering Cancer Centre (MSKCC) Metastatic Colorectal Cancer dataset for CRCs and the MSK-IMPACT Clinical Sequencing Cohort for LACs and MELs. The CRCs included in the MSK-IMPACT Clinical Sequencing Cohort were not adequate because of the lack of MSI data. Each control set was selected based on the following specific criteria: (1) the “gene driver positive” CRC cohort consisted of microsatellite stable (MSS) CRCs with mutations affecting *KRAS*, *NRAS*, and *BRAF* genes for the positions included in our analysis; (2) “gene driver positive” LACs consisted of morphologically defined adenocarcinomas of the selected cohort harbouring mutations in the *EGFR*, *KRAS*, *NRAS*, *BRAF*, and *ERBB2* genes for the positions included in our analysis, and/or and fusions in either *ALK, ROS1*, or *RET* genes; (3) the “gene driver positive” MEL cohort consisted of the melanomas of the selected cohort positive for mutations in the *BRAF* and *NRAS* genes for the positions included in our analysis; and (4) the CRC MSS cohort was defined using the MSI score <10, as previously reported [7]. Cases presenting a *POLE*-dependent profile (*n* = 8) were excluded. For gene rearrangements, we considered only the 14 genes included in the Archer LungFusion Plex Panel (ArcherDx, Boulder, CO, USA). Data were downloaded from the cBioPortal (https://www.cbioportal.org/, accessed on 20 February 2021).

### 2.8. Statistical Analysis

Statistical analyses were performed using GraphPad Prims v.8. The Spearman test was applied to evaluate the correlations between pre-seq and the Archer fusion QC. Fisher’s exact test compared the fusion rates between the “gene driver free” and MSI series and the in silico control series. *p*-values < 0.05 were considered statistically significant.

## 3. Results

### 3.1. Definition of “Gene Driver Free” and MSI Cohorts for Downstream Molecular Analyses

We identified 105 “gene driver free” cases (40 CRCs, 40 MELs, and 25 LACs) and 56 MSI CRCs for a total of 161 patients (Figure 1).

Following pathological review, small LAC samples, MEL micrometastases, and CRCs with a low cellularity were excluded, leading to a final cohort of 125 cases, comprising 70 “gene driver free” and 55 MSI CRCs (Figure 1). Of note, the non-eligibility of these samples stemmed from the small amount of tissue left following previous molecular analyses, and should not be interpreted as non-feasibility of the molecular analysis workflow described in this study. Even if limited, a fraction of these samples could have been analysed, however, we decided to err on a more conservative side, as tissue exhaustion would have most likely occurred.

The majority (60%) of samples belonged to stage III-IV patients, whereas the remaining 40% of samples were predominantly composed of CRCs belonging to the MSI cohort subjected to MSI testing for prognostic stratification, even in the early disease setting (Table 1).

All of the MSI CRCs were wild-type in the target genes, and all of the colorectal cancers’ “gene driver free” were MSS. RNA extraction was successful for all of the cases, with an average yield of 3.4 μg (range: 0.2–7 μg). Two samples showed an Archer PreSeq assay Cq > 31, with values of 31.4 and 32, respectively. The clinico-pathological characteristics of the series are shown in Table 1.

### 3.2. Targeted NGS QC Results Reveal High Feasibility of the Assay in FFPE Samples

The series of 125 cases underwent library preparation, including two RNAs with Archer QC below the established standard and library preparation had a 100% success rate, with only two melanin-rich melanoma samples achieving a suboptimal result (<200 nM). Sequencing on the IonTorrent GeneStudio S5 instrument reached optimal read depth levels for 112/127 (88%). The 15 cases with read depths below 500,000 reads/sample were re-sequenced. The UBAM sequence files produced from these cases were merged, and we obtained a final mean of 813,103 reads/sample (487,000–3,091,921), with a mean on-target of 96%. The mean fusion QC value was 66.64 (range of 8.86–225). The two melanin-rich samples with a fusion QC value below 10 (8.86 and 9.23, respectively) showed a high number of total reads (1,107,457 and 1,146,724) and were included in the subsequent analysis. To evaluate a possible relationship between PreSeq QC and sequencing data, we correlated both the initial quantification and the Ct value of the PreSeq Archer kit, identifying a significant correlation between lower Ct values (e.g., high quality RNAs) and a higher Fusion QC score (Pearson r = −0.30, *p* < 0.001). No correlation was detected between the initial RNA concentration and the Fusion QC score, or between the total reads and the RNA quality control values.

### 3.3. MSI and ”Gene Driver Free” Patients Are Enriched for Fusion Genes, as Detected by the Targeted RNA-Based NGS Panel

**Figure 2:**
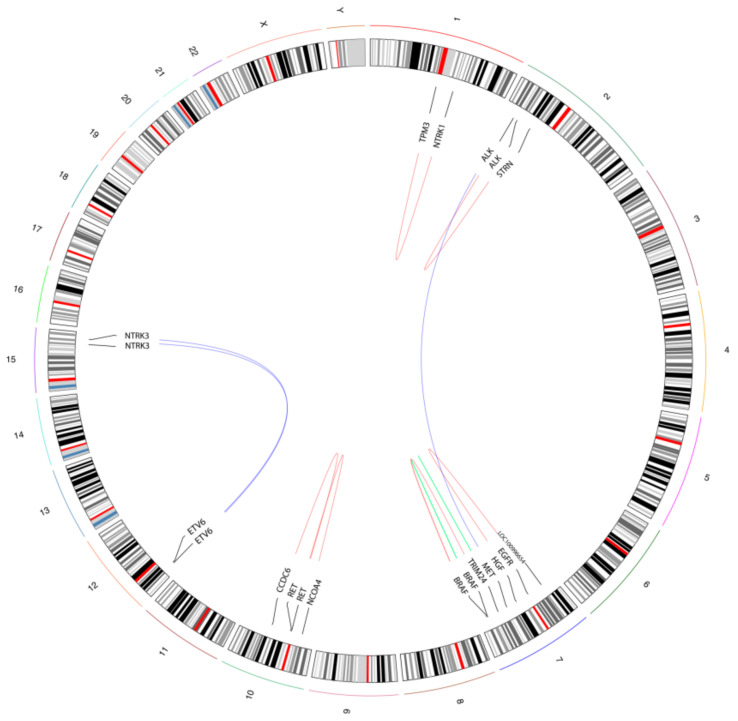
Schematic illustration of the detected gene fusions and rearrangements. The circos plot summarizes the fusion events identified in the “real-world” cohort. The curved lines show partner genes, with blue lines representing inter-chromosomal fusions, red lines indicating intra-chromosomal fusion between different genes, and green lines representing intra-chromosomal rearrangements within the same gene.

We identified 11 gene fusions (8.8%, 11/125), in 7 out of 55 MSI CRCs (12.73%) and in 4 out of 70 of the “gene driver free” population (5.71%), namely three MELs (3/28, 10.7%), and one LAC (1/12, 8.3%), whereas no fusions were identified within the “gene driver free” MSS CRCs (Table 2, Figure 2).

Two fusions involved *NTRK3* with the canonical *ETV6* partner, characterized by two different breakpoints (Figure 2). The *ETV6* ex5→*NTRK3* ex15 was identified in a MSI CRC, whereas the *ETV6* ex4→*NTRK3* ex14 fusion was detected in a LAC. Another *NTRK* gene fusion was identified in an additional MSI CRC, which harboured the *TPM3* ex7→*NTRK1* ex10 (Figure 2). The other gene fusions detected in the MSI CRC cohort included two cases with *ALK* fusions (*HGF* intron16→*ALK* ex2, and *STRN* ex3→*ALK* ex20), 2 cases with *RET* fusions (*CCDC6* ex1→*RET* ex12, *NCOA4* ex9→*RET* ex12) and one case with the *TRIM24* ex3→*BRAF* ex10 fusion (Figure 2). Within the “gene driver free” cohort, in addition to the *ETV6*→*NTRK3* fusion positive LAC, we also detected three rearrangements in three independent melanoma patients: a rare *EGFR* ex26→LOC100996654 fusion, a *BRAF* intradomain duplication between ex10 and ex18 and a *MET* ex14 skipping (Figure 2). The Archer Analysis software also provided an evaluation of frame of the fused RNA: 9 out of 11 rearrangements were predicted to be in frame. The *HGF* intron 16→*ALK* ex2 was predicted to be out of frame, whereas the *EGFR* ex26→LOC100996654 was not predictable, due to the unknown behaviour of the LOC100996654 partner (Table 2).

### 3.4. Enrichment of Gene Fusions in the Study Cohort Is Significantly Higher When Compared to Microsatellite Stable and “Gene Driver Positive” MSKCC Cohorts

**Figure 3:**
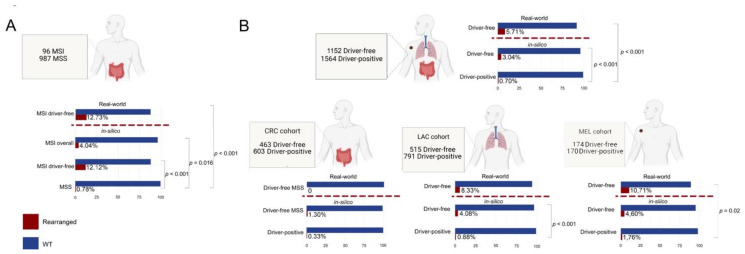
Comparison of the distribution of fusion gene detection rates across “real-world” and in silico cohorts. (**A**) Graphical representation of the in silico microsatellite instable (MSI) cohort and the description of fusion prevalence in both the in silico and the “real-world” MSI cohorts. (**B**) Graphical representation of the cohort as a whole (top) and of the subgroups of cases according to the distinct histology and comparison between “real-world” and in silico data. The percentage of samples harbouring a fusion and significant *p*-values of the contingency tables are shown in a horizontal bar plot. Fusion positive cases are color-coded according to the legend (bottom-left). Dashed red lines separate the study cohort (“real-world”) from the in silico Memorial Sloane Kettering Cancer Centre (MSKCC) cohorts.

We evaluated rates of gene fusions detection in “gene driver free” versus “gene driver positive”, MSS versus MSI patients by interrogating publicly available datasets of the Memorial Sloane Kettering Cancer Centre (in silico MSKCC cohorts). Of note, for the definitions of “gene driver free” and “gene driver positive” in the in silico MSKCC cohorts we considered only mutations in genes and gene positions sequenced for diagnostic purposes, thus allowing comparison with our cohort. Hence, we considered “gene driver free” MSS CRC patients without mutations in *KRAS*, *NRAS* and *BRAF*, LAC patients without *EGFR*, *KRAS*, *NRAS*, *BRAF* and *ERBB2* mutations and with no *ALK*, *ROS1* and *RET* fusions and MEL patients without *BRAF* and *NRAS* mutations. All patients harbouring mutations outside the covered regions were excluded. An MSI score cut-off of 10 was applied to discriminate between the CRC MSI (MSI score > 10) and MSS (MSI score < 10). The in silico cohorts are summarized in Figure 3. In the MSKCC MSI-CRC cohort, 4.04% (4/96) of patients harboured a gene fusion in the included genes, with a significantly higher prevalence compared with the MSS cases (8/987, 0.78%, *p* = 0.016). Similarly, the frequency of gene fusions we detected in our MSI-CRC cohort (12.73%, 7/55) was significantly higher than that observed in the MSKCC MSS-CRC cohort (*p* < 0.0001). Our MSI-CRCs showed a higher prevalence of fusions than the MSKCC MSI-CRC cohort, although the difference was not statistically significant (12.73% versus 4.04%, *p* = 0.09). We observed that the MSKCC MSI-CRC cohort also included patients (64/97, 66%) with either a *BRAF*, *NRAS*, or *KRAS* mutation, whereas our MSI-CRC cohort was entirely composed of *BRAF*/*NRAS*/*KRAS* wild-type tumours. When analysing only the MSKCC CRC cases that were MSI and *BRAF*/*NRAS*/*KRAS* wild-type, the rates of gene fusion detection were superimposable (12.73%, 7/55 in the study cohort and 12.04%, 4/33 in the MSKCC cohort). Interestingly, all of the reported fusions in the MSKCC cohort (*n* = 4) involved an *NTRK* gene (two *NTRK3* and two *NTRK1* fusions), whereas in our series, we detected two *NTRK* gene fusions (one *NTRK3* and one *NTRK1*), two *ALK*, two *RET*, and one *BRAF* gene fusions. Within the context of “gene driver free” patients, 3.04% (35/1152) and 5.71% (4/70) of cases harbouring gene fusions were identified in the MSKCC and in the present cohort, respectively; both rates were significantly higher than those observed in the “gene driver positive” MSKCC cohort (0.70%, 11/1564). Those “gene driver positive” tumours from the MSKCC cohort harbouring a fusion were represented by the following: (i) two CRCs with a *NRAS* p.G13D (*CLVS1*-*FGFR1* fusion) and *BRAF* p.V600E (*BRAF*-intragenic rearrangement), respectively; (ii) two melanomas showing concomitant fusions and driver gene mutations, one with a *BRAF* p.V600E and the *BRAF*-intragenic rearrangement and one with a *NRAS* p.Q61R and an *NTRK1* gene fusion; and (iii) seven LACs patients displaying both driver gene mutations and fusions, of which four harboured the same *EGFR* ex19 deletion (p.E746_A750del), two *KRAS* (p.G12C and p.G12V) mutations, and one *BRAF* p.V600E mutation. Five of these patients carried *BRAF* rearrangements, one showed a *FGFR3* fusion, and one an *NTRK1* gene fusion. When dissecting the “gene driver free” patients according to histology, in our “gene driver free” CRC cohort, which was also MSS, we did not identify any fusions. These types of patients also showed a low frequency of fusions in the MSKCC cohort (1.30%, 6/463), which was not significantly different from the prevalence observed in the CRC “gene driver positive” (0.33%, 2/603 *p* = 0.15). In the “gene driver free” LACs, the fusion detection rate was 8.33% (1/12) in our cohort compared with the 4.08% (21/515) observed in the in silico MSKCC cohort, the latter being significantly higher than the 0.88% (7/791) observed in the “gene driver positive“ LACs of the in silico MSKCC cohort (*p* < 0.001). The difference between our cohort and the MSKCC in silico cohort did not reach significance, most likely due to the low number of LAC cases in our study. The “gene driver free” MEL cases showed a higher frequency of fusion events both in our (10.71%, 3/28) and in the in silico MSKCC cohort (4.60%, 8/174), the first being significantly higher than that of in silico MSKCC MELs (1.76%, 2/170, *p* = 0.02), and the second showing a trend for significance (*p* = 0.07).

### 3.5. Expression of TRK Chimeric Proteins in *NTRK* Fusion Positive Tumours Is Variable, as Detected by Immunohistochemistry

**Figure 4:**
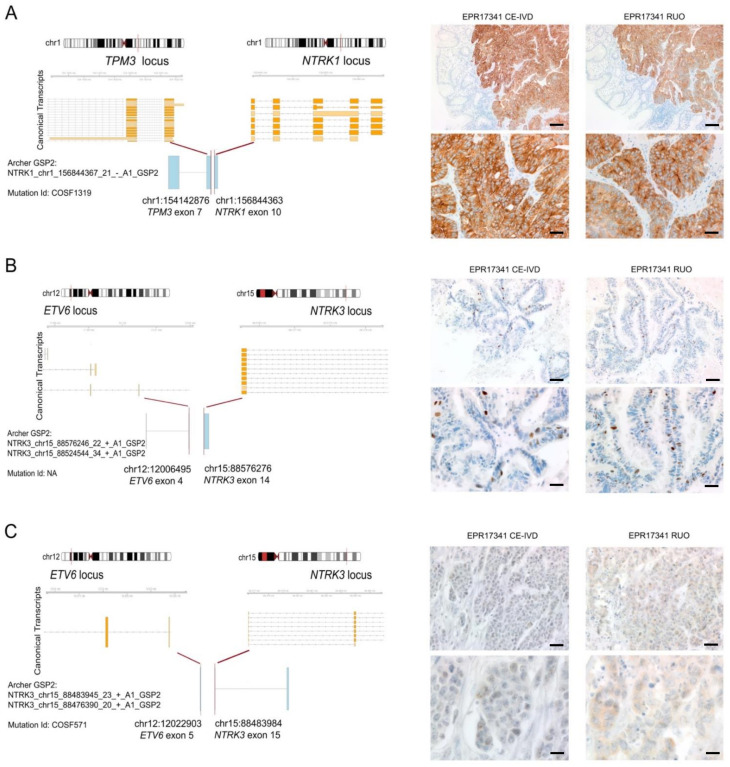
Summary of the detected fusion events involving *NTRK* genes. Each panel consists of a schematic three-tier representation of the fusion identified by the NGS assay and the two pan-TRK antibody IHC stainings. The fusion point is identified by a red bar at the chromosomal loci. Below the chromosomal ideograms, the canonical transcripts reported by Ensembl (release 103) illustrate the transcriptional context of the involved genes (exons are indicated by orange boxes and introns are indicated by grey arrow-headed lines). Each row is a different isoform of the transcript reported by Ensembl. At the bottom, the fusion points between the Archer sequenced exons (blue boxes) and the exact chromosomal position of the breakpoint are reported. (**A**) Representation of the fusion between *TPM3* and *NTRK1* genes. The illustration reports the rearrangement breakpoint, the Archer panel technical output confirming the fusion, and the COSMIC ID. Representative micrographs of the CRC sample harbouring the fusion show a diffuse cytoplasmic expression with focal membranous linear patterns in the experiments run with both CE-IVD and RUO assays (scale bars: top panels, 100 μm; bottom panels, 50 μm). (**B**) Representation of the fusion between the *ETV6* ex4 and *NTRK3* ex14 genes. The illustration reports the rearrangement breakpoint, the Archer panel technical output confirming the fusion, and the COSMIC ID. IHC images highlight the presence of a subpopulation of tumour cells of the LAC with papillary features displaying an intense nuclear expression, as demonstrated by both assays (scale bars: top panels, 100 μm; bottom panels, 50 μm). (**C**) Representation of the fusion between *ETV6* ex5 and *NTRK3* ex15 genes. The illustration reports the rearrangement breakpoint, the Archer panel technical output confirming the fusion, and the COSMIC ID. A weak cytoplasmic expression is present in this CRC in both experiments, with the RUO assay showing a slightly more granular staining (scale bars: top panels, 50 μm; bottom panels, 25 μm).

For the *NTRK1/2/3* gene fusion detection, we also investigated two alternative methods, i.e., multi-target qRT-PCR and IHC using both the CE-IVD/class I US analytical assay pan-TRK by Roche-Ventana and the RUO pan-TRK antibody by Abcam. These two assays share the same antibody clone (EPR17341). The success rate of qRT-PCR was 98.4% (123/125). Cases with test failure were MELs rich in melanin pigment. All of the identified fusions were confirmed in two independent experiments. IHC scoring was performed using a highly conservative approach, as suggested by international recommendations [9], as follows: any detectable staining in cancer cells, even when focal and/or faint, was considered as a possible positivity of the assay. Accordingly, we detected specific staining in 13/125 cases (10.4%), with overall concordant results between the two assays. Out of the 13 cases showing a TRK expression, three (23%) harboured an *NTRK* fusion: these corresponded to one LAC harbouring an *ETV6*→*NTRK3* and two CRCs, harbouring the *TPM3*→*NTRK1* and the *ETV6*→*NTRK3* fusions, respectively (Figure 4).

**Figure 5:**
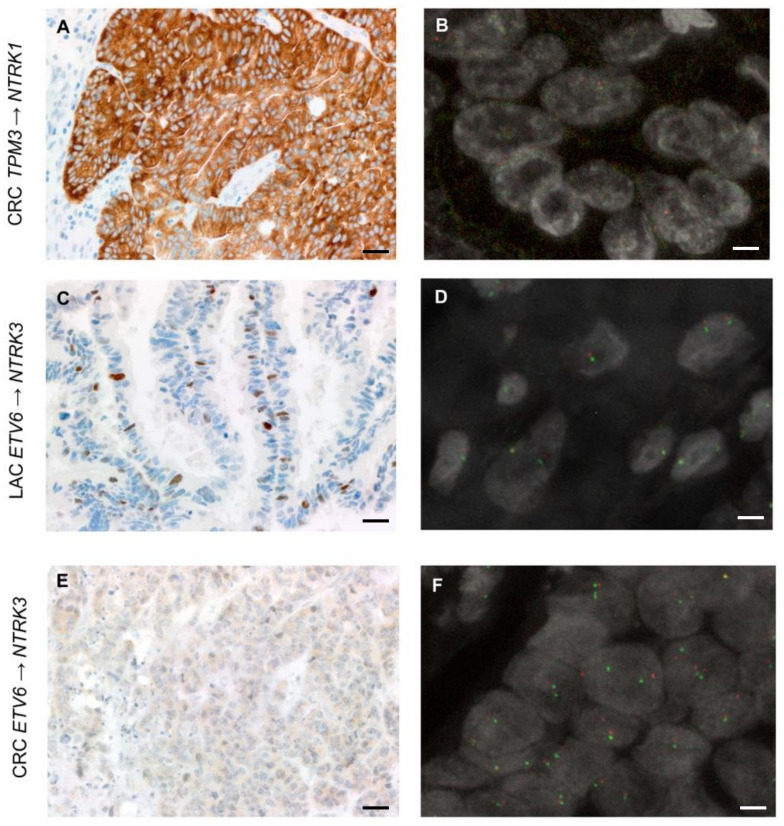
Representative IHC and FISH images of the cases identified as harbouring an *NTRK* gene fusion (scale bars—IHC images: 50 μm; scale bars—FISH images = 5 μm). (**A**,**B**) The FISH test for the CRC harbouring the intrachromosomal *TPM3*→*NTRK1* rearrangement, as identified by NGS, and displaying strong and diffuse panTRK expression by IHC was suboptimal in terms of quality. Following multiple attempts in all of the available tumour blocks, in scattered tumour cells showing red and green signals, the NTRK1 probes appeared slightly split-apart. This FISH test would have led to an indeterminate result in a diagnostic setting. (**C**,**D**) In the LAC sample harbouring the *ETV6*→*NTRK3* fusion, occasional (<10%) nuclei with signals pertaining to NTRK3 probes that were slightly split-apart could be identified. It should be acknowledged that according to standard FISH scoring criteria, (i) the distance between signals may be debatable and (ii) the test would be considered negative, given that the positive nuclei do not reach the 15% cut-off used in the diagnostic setting. (**E**,**F**) The CRC sample harbouring the *ETV6*→*NTRK3* fusion showed split-apart NTRK3 signals in about 17% of the tumour cell population (here illustrated). This FISH test meets the criteria for the identification of a genetic rearrangement involving *NTRK3*.

The *TPM3*→*NTRK1* CRC showed strong and diffuse cytoplasmic staining in >80% of tumour cells, featuring occasional linear staining along the membrane (Figure 4A). The *ETV6*→*NTRK3* LAC showed an intense but focal nuclear expression (<10% of tumour cells) (Figure 4B). The *ETV6*→*NTRK3* CRC showed faint cytoplasmic staining identified in a subpopulation of 30% of tumour cells in the RUO assay, and faint/barely perceptible cytoplasmic staining in about 30% of tumour cells when using the CE-IVD assay (Figure 4C). These results are in line with the number of unique starting sites (SS) detected by the NGS panel—the fusion involving *NTRK1* was identified by 72 unique SS reads, whereas the two less expressed fusions of *NTRK3* by 23 (*ETV6*→*NTRK3* in the LAC) and 33 (*ETV6*→*NTRK3* in the CRC) unique reads (Table 2). Based on these data, the sensitivity of IHC for both antibodies was 100%, and the specificity was 91.7%. Tumours showing TRK expression without evidence of an *NTRK* fusion according to the molecular assays used displayed faint cytoplasmic staining in most of the cases (8/10), whereas two cases displayed focal expression (<10% of tumour cells) of a moderate intensity, either cytoplasmic or nuclear. In the *NTRK* gene fusion positive cases, we also performed FISH analysis with the specific probes for the *NTRK* gene involved in the fusion (Figure 5). In the CRC sample harbouring the *ETV6*→*NTRK3* fusion and showing faint/barely perceptible IHC staining, FISH with *NTRK* probes revealed a subpopulation of cells (quantified as 17%) harbouring split-apart signals. More challenging was the *ETV6*→*NTRK3* fusion positive LAC, where occasional nuclei with signals that were slightly apart could be observed, even though we acknowledge that the distance separating the signals was borderline according to the standard FISH scoring criteria. In addition, the latter FISH pattern would be consistent with a negative result according to the 15% cut-off conventionally applied in diagnostic FISH testing for gene fusion detection. Finally, the FISH test performed on the *TPM3*→*NTRK1* fusion positive CRC showing a diffuse and strong pan-TRK expression resulted in suboptimal quality following several attempts on multiple tumour blocks: only occasional nuclei presented signals, which were slightly split-apart; nevertheless, the test would be scored as indeterminate in diagnostic practice.

## 4. Discussion

Here, we report a significant number of gene fusions detected by a relatively small, targeted RNA-based NGS panel (14 genes) in a real-world cohort of advanced stage patients affected by colorectal cancer, melanoma, or lung adenocarcinoma proven to be wild-type by diagnostic hotspot mutational analysis for actionable alterations, or with a diagnosis of MSI colorectal carcinoma. In addition, we provide comparative data for distinct techniques employed for *NTRK* gene fusion detection, and show the following: (i) 100% sensitivity of immunohistochemistry, yet challenging patterns to be recognized in order not to miss a possible gene fusion; (ii) good performance of multiplex qRT-PCR, even though not all of the samples could be successfully analysed; and (iii) an excellent performance of an RNA-based NGS panel, even when the pre-sequencing quality control was suboptimal for a relatively small subgroup of samples. 

We have recently witnessed the detection of specific genetic alterations in a histology-independent fashion, leading to the establishment of tumour agnostic biomarkers. MSI has pioneered this field; as a predictor of the response to immunotherapy, it led to the first agnostic approval (pembrolizumab treatment) in 2017 [25]. More recently, gene fusions involving kinases have been described across several malignancies. Among these, *NTRK* gene fusions represent the boldest example overall, with a prevalence of 0.3% in a plethora of common malignancies and high frequencies (>95%) in a handful of rare histologies [9]. *NTRK* gene fusion positive tumours have also provided successful examples of novel targeted therapies in the clinical setting, with substantial and durable responses documented for first generation TRK inhibitors, which led to FDA and EMA approvals of larotrectinib and entrectinib for patients at an advanced stage [26]. Moreover, several trials targeting gene fusions are on-going, e.g., in the case of *FGFR1/2/3*, *ROS1*, *ALK*, and *RET* genes [27]. The RET inhibitor selpercatinib has recently been approved for lung and thyroid cancers harbouring *RET* gene mutations or fusions [10], and pralsetinib has been recently reported to be a new, well-tolerated, promising treatment option for patients with *RET* fusion-positive LACs [28]. Other examples of rare, yet actionable genetic alterations in more than one histology are offered by rearrangements of the *MET* (skipping of exon 14) and *BRAF* genes. Retrospective studies and clinical trials have shown that *MET* exon 14 skipping alterations confer sensitivity to MET inhibitors (crizotinib or capmantinib) [29]. A more complex scenario is reported for *BRAF* rearrangements: indeed, although both the V600E variant and the non-V600E mutations are approved targets for LACs, CRCs, and MELs [30], little is known about *BRAF* fusions and intra-domain duplications. Kinase domain duplications are reported as a mechanism of resistance to classical BRAF inhibition; however, lesions harbouring this alteration have been proven to be sensitive to the pan-RAF inhibitor LY3009120 [31]. Different fusion partners have also been recently shown to confer heterogeneous oncogenetic potentials to *BRAF* fusions [32]. In particular, in organoid models of CRCs, *TRIM24-BRAF* fusions conferred resistance to EGFR and MEK inhibition, whereas substantial sensitivity to RAF and ERK inhibition was reported both in single and combined therapy [32].

In this context, the challenge for diagnostic practice is two-fold: (i) to cope with the low rate of detection of these alterations across common malignancies and (ii) to re-think molecular testing based more likely on the genetic alteration rather than the site of origin of the tumour. Hence, major efforts are currently being put in place to identify a subgroup of patients who may be the most likely carriers of these rearrangements [14,15]. Our study demonstrates a high frequency of targetable kinase fusions in tumours lacking a concomitant alteration in canonical driver genes, or with a background of instability of the microsatellite sequences when in the context of CRC. The correlation between the enrichment of gene fusions and MSI CRCs has been previously reported. Cocco and co-workers [16] reported a 5% fusion detection in MSI/MMR-deficient colorectal carcinoma and 15% MSI/MMR-deficient colorectal carcinoma with wild-type *RAS/BRAF*. Similarly, Sato et al. [20] identified 11 fusion kinases in MSI CRCs that lacked oncogenic *KRAS/BRAF* missense mutations. Of note, both studies observed that kinase fusions were associated with sporadic MSI CRC and with *MLH1* promoter hypermethylation status [16,20]. These data are in line with our sporadic series of MSI CRCs, which were all *RAS/BRAF* wild-type. It appears, therefore, that in CRCs, mutual exclusivity between mutations in the main MAPK genes and gene fusion events happens in the context of an MSI background [16,20]. Indeed, no significant gene fusion enrichment was observed in “gene driver free” MSS patients, as identified in our cohort and in the in silico MSKCC datasets re-analysed here. With respect to the degree of mutual exclusivity between mutations in cancer associated driver genes and fusion events across distinct histologic types, a pioneering in silico work analysing nearly 9000 cases of the TCGA cohort from 33 different tumour types identified 299 driver genes, with few patients with concomitant mutations and fusions in cancer-related genes (around 6%, when considering variants affecting also tumour suppressor genes) [15]. The rare simultaneous detection of chimeric transcripts in driver genes and mutations of the MAPK pathway has also been documented in patients with melanoma, pancreatic, and lung cancers [17,18,19]. Of note, all of the studies reporting on this mutual exclusivity were based on wide genomic screening, with a declared discovery research approach. Although a comprehensive genomic profiling of tumours would be informative from the beginning for advanced stage patients, its large-scale applicability in clinical diagnostic practice is still questionable. Nevertheless, clinical sequencing can be exercised at different levels of complexity. In this study, we aimed to downscale this approach to a minimum number of genes analysed in the clinical setting with the implementation of an additional panel, which was rather small yet informative with respect to the current FDA/EMA approvals. We were able to demonstrate that rare alterations inducing the generation of chimeric transcripts are significantly increased in LACs, MELs, and MSI-CRCs that have a wild-type result for the limited number of genes currently analysed to decide standard of care first line treatment. The proven feasibility of the method and the identification of these “enriched populations” encourage the pursuit of gene fusions in this context. Data were provided across three different malignancies, thus paving the way for an agnostic process that is centred on the genetic alterations and feeds distinct histologic types. Given the complexity of *NTRK* gene fusion detection, which involves three different genes, we also sought to investigate the performance of different testing strategies that can be easily applied in diagnostic practice. All of the *NTRK* gene fusions identified in our series by targeted RNA-based NGS have been previously reported and are part of the 32 known *NTRK* fusions included in the qRT-PCR assay. This is the reason for the 100% sensitivity of this assay, which per se has a limited reference range. Nevertheless, three melanoma samples particularly rich in melanin failed to be analysed by qRT-PCR, whereas they showed acceptable results with RNA-based NGS. The ability of NGS to identity chimeric transcripts has been widely described and debated. In a recent paper [33], commercially available multitarget sequencing methods were compared in order to evaluate *NTRK1/2/3* fusions, and the authors reported a strong concordance across different tests. Heyer and colleagues [34] described an increase in the fusion diagnostic rate from 63% to 76% compared with mono-target FISH and qRT-PCR. Immunohistochemistry represents a possible screening tool in the context of the so called “two-step approach”, where a reflex molecular test is mandatory in order to ascertain whether the detected expression stems from the presence of an *NTRK* gene fusion [9]. Indeed, the TRK inhibitor prescription relies on the identification of *NTRK* gene fusion at a molecular level. In our study, we report a 100% sensitivity and 91.7% specificity for this technique. *NTRK* expression was variable in terms of pattern, intensity, and pervasiveness across the tumour cell population. The expression detected in tumours that were proven *NTRK* wild-type featured preferably faint and/or barely perceptible staining; however, two cases showed nuclear or cytoplasmic expression of a moderate intensity. A strong TRK expression was only observed in two cases harbouring an *NTRK* gene fusion. The *TPM3-NTRK1* fusion positive CRC displayed strong and diffuse cytoplasmic staining with focal membrane enhancement, whereas the *ETV6-NTRK3* fusion LAC showed strong nuclear expression in a subpopulation (10%) of tumour cells. Finally, the *ETV6-NTRK3* fusion positive CRC demonstrated faint/barely perceptible cytoplasmic staining, which was too subtle to be detected at first glance. Of note, tumours harbouring *NTRK3* fusions have been previously shown to have much weaker and more focal staining for pan-TRK than tumours with *NTRK1/NTRK2* fusions [9,35], and a lower sensitivity of pan-TRK assays has been specifically reported for *NTRK3* fusions [9,35], which also typically show nuclear expression patterns [9,35]. Taken together, these results support the recommendation of considering any type of staining whenever IHC is used as a screening tool [9], and suggest being very conservative in the assessment of this assay. Nevertheless, the detailed comparative data we report may open questions on the response to TRK inhibitors. The expression of the protein product of the *NTRK* chimeric gene is of particular importance because it represents the pharmacological target. It remains to be determined whether the variable and possibly heterogeneous TRK expression that we document here may result from fusions that did not effectively or pervasively result in the translation of the protein and whether this may correspond to differential response to TRK inhibitors. Of note, the retrospective evaluation of our NGS data of *NTRK* fusion positive tumours confirmed this heterogenous expression: the absolute quantity of the *NTRK* chimeric transcripts, calculated as the number of unique reads, was highly variable within the positive samples, and correlated with both the qRT-PCR and IHC results. Finally, we also tested the FISH analysis, even though it was uniquely used as a confirmatory methodology in those cases identified as positive by NGS and IHC. The output of this technique was challenging in terms of performance and interpretation, thus further discouraging its implementation in the context of *NTRK* gene fusion testing.

## 5. Conclusions

In conclusion, we have demonstrated a strategic workflow for enriched gene fusion detection in cancer patients, easily applicable to the clinical setting, at first diagnosis, and over the treatment course. This was demonstrated in three different malignancies, thus paving the way for an agnostic process centred on genetic alteration to pursue feeding distinct malignancies to be investigated. Although mutations in canonical driver cancer genes and gene fusions are not entirely mutually exclusive events, there is a clear enrichment of chimeric genes in tumours that have been proven to be wild-type following the essential molecular screening currently offered to all advanced stage patients. Similarly, MSI CRC patients are worth additional investigation. Our comparative data specific to *NTRK* gene fusion detection show the feasibility of RNA-based NGS targeted panels on FFPE samples, and the variable TRK expression in *NTRK* fusion positive tumours, thus voicing a word of caution whenever interpreting this assay and opening questions related to the correlation with response to TRK inhibitors, which remains to be determined.

Our study focussed on a pragmatic approach to detect actionable gene fusions in a “real-world” clinical setting, exploiting a relatively small but informative panel and correlation with previous molecular results. This has the advantage of providing a feasible and easily accessible additional test with enrichment in the detection of actionable genetic alterations. Nevertheless, we should acknowledge that the use of large NGS panels leading to a comprehensive genomic profiling of tissue and/or liquid biopsies would represent a further level of informativeness for unselected patients at an advanced disease stage, even with respect to a higher likelihood of decoding possible mechanisms behind therapy resistance. Major drawbacks in this scenario relate to test accessibility and the accurate management and interpretation of output data, best handled within multidisciplinary molecular tumour boards.

## Figures and Tables

**Table 1:** Clinico-pathological features of the patient series.

Age (Range)		CRC		LAC	MEL
		MSI	Driver-Free		
		68 (26–88)	62 (37–80)	66 (55–78)	65 (37–90)
Gender	Male	23	21	9	14
	Female	32	9	3	14
	Total	55	30	12	28
Specimen type	Resection	55	23	8	15
	Biopsy	0	6	2	13
	Cytologic sample	0	1	2	0
	Total	55	30	12	28
Lesion type	Primary tumour	55	21	10	21
	Metastatic deposit	0	9	2	7
	Total	55	30	12	28
Stage	I	14	0	3 *	0
	II	27	3	0	2
	III	11	10	2	12
	IV	3	17	7	14
	Total	55	30	12	28

MSI—microsatellite instability; CRC—colorectal cancer; LAC—lung adenocarcinoma; MEL—melanoma. * Molecular test performed on second primary tumours.

**Table 2:** Summary of detected gene fusions.

ID	Tumour Type	Cohort	Tumour Cell Content (%)	Rearrangement	# READS	Reading Frame
4_DRIVER FREE	MEL	Driver Free	90	*BRAF* Domain Duplication	258	In frame
53_DRIVER FREE	MEL	Driver Free	80	*EGFR* ex26→LOC100996654	45/67	n.a.
34_MSI	CRC	MSI-CRC	60	*TPM3* ex7→*NTRK1* ex10	72/513	In frame
11_DRIVER FREE	LAC	Driver Free	70	*ETV6* ex4→*NTRK3* ex14	23/23	In frame
1_MSI	CRC	MSI-CRC	50	*ETV6* ex5→*NTRK3* ex15	33/45	In frame
3_MSI	CRC	MSI-CRC	80	*HGF* intron16→*ALK* ex2	10/21	Out of frame
48_DRIVER FREE	MEL	Driver Free	80	*MET* ex14 skipping	342	In frame
28_MSI	CRC	MSI-CRC	50	*STRN* ex3→*ALK* ex20	78/252	In frame
53_MSI	CRC	MSI-CRC	70	*TRIM24* ex3→*BRAF* ex10	61/170	In frame
55_MSI	CRC	MSI-CRC	80	*CCDC6* ex1→*RET* ex12	122/784	In frame
44_MSI	CRC	MSI-CRC	70	*NCOA4* ex9→*RET* ex12	54/68	In frame

MSI—microsatellite instability; CRC—colorectal cancer; LAC—lung adenocarcinoma; MEL—melanoma; n.a.—not assessable.

## Data Availability

The main research data supporting the results of this study are included in Table 1 and Table 2 and Figure 1 and Figure 5. Other data can be made available upon reasonable request from the corresponding author.
